# Oxidized Bacterial Cellulose Membranes Immobilized with Papain for Dressing Applications: Physicochemical and In Vitro Biological Properties

**DOI:** 10.3390/pharmaceutics16081085

**Published:** 2024-08-18

**Authors:** Niédja Fittipaldi Vasconcelos, Pascale Chevallier, Diego Mantovani, Morsyleide de Freitas Rosa, Fernando José Soares Barros, Fábia Karine Andrade, Rodrigo Silveira Vieira

**Affiliations:** 1 Centro de Tecnologias Estratégicas do Nordeste (CETENE), Laboratório de Materiais Nanoestruturados (LMNano), Cidade Universitária, Avenida Professor Luiz Freire 01, Recife 50740-540, PE, Brazil; 2 Laboratory for Biomaterials & Bioengineering (LBB), Department of Min-Met-Materials Engineering & CHU de Quebec Research Center, Laval University, Quebec, QC G1V 0A6, Canada; pascale.chevallier@crchudequebec.ulaval.ca (P.C.); diego.mantovani@gmn.ulaval.ca (D.M.); 3 Embrapa Agroindústria Tropical–CNPAT, Rua Dra Sara Mesquita 2270, Planalto do Pici, Fortaleza 60511-110, CE, Brazil; morsyleide.rosa@embrapa.br; 4 Departamento de Engenharia Química, Universidade Federal do Ceará (UFC), Bloco 709, Fortaleza 60455-760, CE, Brazil; fernando_barros17@yahoo.com.br (F.J.S.B.); fabiakarine@gmail.com (F.K.A.); rodrigogpsa@gmail.com (R.S.V.)

**Keywords:** hemocompatibility, cytotoxicity, fibroblasts, keratinocytes, debridement, dressing

## Abstract

This research consolidates our group’s advances in developing a therapeutic dressing with innovative enzymatic debridement, focusing on the physicochemical and in vitro biological properties of papain immobilized in wet oxidized bacterial cellulose (OxBC–Papain) dressing. OxBC membranes were produced with *Komagataeibacter hansenii* oxidized with NaIO_4_, and papain was immobilized on them. They were characterized in terms of enzyme stability (over 100 days), absorption capacity, water vapor transmission (WVT), hemocompatibility, cytotoxicity, and cell adhesion. The OxBC–Papain membrane showed 68.5% proteolytic activity after 100 days, demonstrating the benefit of using the OxBC wet membrane for papain stability. It had a WVT rate of 678 g/m^2^·24 h and cell viability of 99% and 86% for L929 and HaCat cells, respectively. The membranes exhibited non-hemolytic behavior and maintained 26% clotting capacity after 1 h. The wet OxBC–Papain membrane shows significant potential as a natural biomolecule-based therapeutic dressing for wound care, offering efficient debridement, moisture maintenance, exudate absorption, gas exchange, and hemostasis without cytotoxic effects or cell adhesion to the dressing. Further research, especially using in vivo models, is needed to assess its efficacy in inducing epithelialization. This study advances stomatherapy knowledge, providing a cost-effective solution for enzymatic debridement in healthcare.

## 1. Introduction

Skin wound care has become an important medical approach within the healthcare system due to the high psychological, physical, and financial burdens on both patients and hospitals [1]. Physiologically, the healing process can be divided into four distinct and consecutive phases: hemostasis, inflammation, cell proliferation, and tissue remodeling [2]. The treatment of skin wounds is dynamic and depends, at all stages, on the evolution of the healing phases. Effective management of the healing phases is necessary to avoid complications and prevent the worsening of the patient’s condition in chronic wounds, such as severe burns, pressure ulcers (decubitus), and venous ulcers. These wounds have difficulty in regenerating epithelial and granular tissue due to the high amount of pro-inflammatory cytokines [3,4,5,6,7].

A small cut generally recovers within a few days thanks to the body’s strong orchestration, which promotes cell migration and maintains adequate levels of inflammation, innervation, and angiogenesis. Larger injuries may take several weeks to heal, eventually leaving a visible scar. However, chronic wounds cannot follow the physiological steps to recover the tissue in a short period; they can last for more than three months and even years. This makes treating them a major therapeutic challenge worldwide [4].

The appropriate strategy for the treatment of chronic skin wounds mainly includes the application of dressings and external therapeutic methods, such as negative pressure therapy and hyperbaric oxygen therapy. These treatments are closely associated with a deep and detailed knowledge of the healing process, the intrinsic factors involved, and the local conditions affecting the wound [1,8,9,10,11].

With regard to the application of dressings, their intrinsic properties need to be assessed, including the morphology (microscopy), the water and body fluid absorption capacity (swelling), hydrophilicity (contact angle analysis), water vapor transmission (WVT) and oxygen transmission (O_2_), elasticity and tensile strength (mechanical tests), antimicrobial activity, and drug release profile (when a bioactive agent is intended to be incorporated into the dressing), and in vitro and in vivo cytotoxicity tests also need to be conducted [12,13,14,15,16].

In this context, the use of bacterial cellulose (BC) as a dressing has been of great interest due to its favorable characteristics, such as biocompatibility, non-toxicity, chemical stability, mechanical resistance, and high water content [17,18,19,20,21]. The continuous excretion of cellulose by bacteria results in a wet membrane with a natural porosity gradient. The first layers formed are more compact and unified, while the subsequent layers are more widely spaced, creating a porous structure throughout the thickness of the membrane. This porosity can also be improved using a purification process via alkaline treatment with potassium carbonate (K_2_CO_3_) [22,23]. Then, when applied as a dressing, this highly porous cellulose membrane begins to act as a “sieve”, controlling gas exchange, maintaining an appropriate level of humidity, preventing bacterial penetration, absorbing excess exudate, and regulating the temperature [3,19,21,24]. The other advantages of BC materials are their flexibility, ease of application, painless removal, low cell adhesion (preventing further tissue damage when changing dressings), and high thermal stability (sterilization by autoclaving). Furthermore, the use of a wet BC membrane has the advantage of rapidly relieving damaged skin and hydrating tissues (especially in the case of burns and/or frostbite) [24].

The aforementioned features offer more suitable conditions that are more conductive to tissue regeneration than traditional dressings, such as cotton, synthetic and natural gauzes, and bandages. Older approaches to wound dressings have varying degrees of absorption that can cause incomplete tissue growth and new injuries after dressing removal [25]. Therefore, a BC membrane is considered a promising naturally structured material for passive wound dressings. However, despite BC having intrinsic characteristics that encourage its use as a dressing, there are not many commercial products based on BC. BC manufacturers are mainly located in the United States of America, Brazil, and Poland [26]. Some of the commercial dressings are Membracel, commercialized by Vuelo Pharma (Curitiba, Brazil), EpiProtect^®^ from S2Medical AB (Linköping, Sweden), Celmat from Bowil Biotech (Władysławowo, Poland), and Axcelon Biopolymers Corp (London, Canada). BC dressings containing bioactive compounds are even less commercially explored, for example, Nanoderm^TM^ Ag from Axcelon Biopolymers Corp (London, Canada), which releases silver for the treatment of infected wounds, and Suprasorb^®^ X + PHMB from L&R Company (Lohmann & Rauscher, Rengsdorf, Germany), which contains poly hexamethylene biguanide, a cationic polymer with antimicrobial and antiviral properties.

A wet BC membrane has been used strategically by our research group to obtain a heterofunctional immobilization support that is capable of adequately immobilizing papain through the association of chemical (by covalent bond) and physical (electrostatic interaction) methods [23,27]. This multifunctionality of the wet oxidized bacterial cellulose membrane (OxBC) favors a rapid interaction (chemical and ionic) of papain, allowing it to act as a debriding therapeutic dressing on necrotic and chronic wounds, enhancing the healing process.

The use of papain as a bioactive agent in a wound is justified because it is a proteolytic enzyme (derived from the latex of *Carica papaya*), which has garnered significant attention for its diverse applications across various fields, particularly in stomatotherapy. Historically utilized in pharmaceutical forms, such as powders, gels, and creams, papain has demonstrated high efficacy in removing necrotic tissues (debridement action) from the wound bed, accelerating the healing process of chronic wounds and foot ulcers [28]. Despite these benefits, the direct application of papain can be challenging due to its potential deactivation under adverse environmental conditions, which has led to innovations in enzyme immobilization on polymeric supports to enhance stability and efficacy [20,27,29,30]. Therefore, optimizing the pH and temperature parameters to enable high proteolytic activity in papain was validated by our research group to obtain a bioactive dressing with debridement properties that can be usefully applied to acute and chronic skin wounds [27]. Investigating the physicochemical and in vitro biological properties of bioactive dressing developed from the combination of OxBC and papain (OxBC–Papain) is essential for validating the procedure established by our research group. This investigation ensures the robustness and reliability of the scientific data obtained and provides crucial information for future research, particularly using in vivo models, to confirm the efficacy of the bioactive dressing.

Recent advancements have explored the incorporation of papain into cellulose-based membranes for wound dressings, utilizing various immobilization techniques to maintain its proteolytic activity while minimizing cytotoxicity. Traditional methods involving toxic spacers, like glutaraldehyde, have raised concerns [21]. In contrast, newer approaches using succinic acid, EDC (1-ethyl-3-3-dimethylaminopropylcarbodiimide), and NHS (N-hydroxysuccinimide) offer a safer alternative, preserving the structural integrity of the bacterial cellulose and ensuring controlled enzyme release [20]. A highlight of innovation for the use of bacterial cellulose as a support for papain immobilization is its wet use, as a hydrogel, which was proposed by our research group [27]. The water intrinsic to the BC membrane favors the proteolytic activity of papain, namely enzyme release, and prevents tissue dryness and discomfort in the patient. As an antibacterial agent, the specificity of papain towards dead tissue facilitates effective wound debridement and accelerates tissue regeneration, positioning it as a valuable component in modern wound care products [21]. These developments reflect a growing recognition of the potential of papain in enhancing patient comfort and treatment outcomes, driving ongoing research and innovation in its applications [20,21,27,29].

This research focuses on the advances made by our group in developing a therapeutic dressing with an innovative enzymatic debridement profile. Consequently, this article aims to consolidate the scientific data previously obtained by our group and to determine the physicochemical and in vitro biological properties of the OxBC–Papain wet membrane. The goal is to contribute to the field of stomatherapy, which is dedicated to the treatment of wounds.

## 2. Experimental Procedure

### 2.1. Reagents

Calcium chloride (CaCl_2_, code 21115), casein from bovine milk (code C3400), citric acid (code 251275), high-glucose Dulbecco’s Modified Eagle’s Medium (code D0822), EDTA disodium salt (C_10_H_14_N_2_Na_2_O_8_∙2H_2_O, code E4884), ethylene glycol (C_2_H_6_O_2_, code 324558), fetal bovine serum (FBS, code F1051), hydrochloric acid (HCl, code 320331), L-cysteine hydrochloride monohydrate (C_3_H_7_NO_2_S∙HCl∙H_2_O, code C7880), Penicillin-Streptomycin (code P4333), nutrient agar (code 70148), phosphate-buffered saline (PBS, code P4417), potassium carbonate (K_2_CO_3_, code P1472), potassium chloride (KCl, code 60130), resazurin (C_12_H_6_NNaO_4,_ code R7017), saponin (code 558255), sodium periodate (NaIO_4_ code 51878), and trichloroacetic acid (C_2_HCl_3_O_2_, code T6399) were purchased from Sigma-Aldrich^®^ (Oakville, ON, Canada) and Merck^®^/Sigma-Aldrich^®^ (Barueri, Brazil). Pure papain (code P.10.0800.000.00) was purchased from Dinâmica Química Contemporânea Ltd.a, Indaiatuba, Brazil. All of the analytical-grade reagents were used without further purification as received from the suppliers. *Komagataeibacter hansenii* (*K. hansenii* ATCC 53582) and normal human dermal fibroblasts (HDF, CRL-2565) were obtained from the American Type Culture Collection (ATCC/EUA). L929 cells (code 0188) and HaCat cells (code 0341) were obtained from Banco de Células do Rio de Janeiro (BCRJ, rep ATCC/Brazil).

### 2.2. Methods

The procedures for producing and purifying the BC membrane and preparing the oxidized bacterial cellulose (OxBC) membrane followed the methodology described in our previous works [23,27].

#### 2.2.1. Production and Purification of BC Membranes

*Komagataeibacter hansenii* (ATCC 53582) produced BC membranes via static fermentation. The culture medium, as described by Hestrin and Schramm [31], was prepared with 20 g·L^−1^ glucose, 5 g·L^−1^ peptone, 5 g·L^−1^ yeast extract, 1.15 g·L^−1^ citric acid, and 2.7 g·L^−1^ Na_2_HPO_4_ and adjusted to pH 5 with 0.1 mol·L^−1^ NaOH or HCl. The culture medium was autoclaved at 121 °C for 20 min and then inoculated with 3% (*v*/*v*) *K. hansenii*. BC membranes were produced in 500 mL Schott bottles (86 × 181 mm) containing 120 mL of culture medium and incubated statically in a refrigerated incubator (BOD TE 391, Tecnal, Piracicaba, SP, BR) at 30 °C for 5 days.

For purification, BC membranes were washed with tap water, followed by immersion in distilled water, and heated at 80 °C for 1 h. This procedure was repeated twice to eliminate excess culture medium and some of the microbial content. Subsequently, the BC membranes were treated twice with 0.3 mol·L^−1^ K_2_CO_3_ at 80 °C for 1 h to ensure the complete removal of bacteria and residual culture medium. Finally, the purified BC membranes were washed with deionized water at 25 °C until reaching a neutral pH and autoclaved for 20 min at 121 °C for sterilization.

#### 2.2.2. Preparation of Oxidized BC Membrane (OxBC)

For membrane oxidation, the purified BC was pretreated by immersion in KCl/HCl solution (pH = 1) at 25 °C for 24 h. Then, 17 g of wet BC membrane was immersed in 140 mL of 1% (*w*/*v*) NaIO_4_ solution prepared in the same KCl/HCl solution. The oxidation reaction occurred at 55 °C for 6 h in a shaking incubator (TE-420, Tecnal, Piracicaba, SP, Brazil) with orbital shaking (125 rpm) and the absence of light. After the end of the reaction, the excess NaIO_4_ was neutralized by the addition of 12 mL of ethylene glycol, and the system was kept for an additional hour at 25 °C under orbital shaking (125 rpm). Then, the OxBC membrane was washed several times with deionized water to eliminate any residual reagents and sterilized in an autoclave for 15 min (121 °C; 1 atm).

#### 2.2.3. Production of OxBC–Papain Membrane

The bioactive dressing was produced by papain immobilization using a wet and sterile OxBC membrane (~16 g wet weight and thickness of 3.7 mm). Previously, the papain solution (2%, *w*/*v*) was prepared in 0.1 mol·L^−1^ citrate-phosphate buffer (pH = 7) and sterilized by filtration in a 0.22 μm polyethersulfone (PES) filter. Wet and sterile OxBC membrane was immersed in 25 mL (500 mg papain powder) of sterile papain solution using sterile polystyrene Petri dishes (150 mm diameter × 25 mm height). The entire procedure described was carried out in a laminar flow cabinet to guarantee a contamination-free environment. The Petri dishes were tightly closed and incubated in an oven at 45 °C for 24 h. At the end of the immobilization process, the wet OxBC membrane containing papain (OxBC–Papain) was washed with 25 mL of sterile phosphate-buffered saline (PBS 0.1 mol·L^−1^, pH = 8) to remove the non-immobilized enzyme. Finally, the OxBC–Papain membrane was stored in a sterile Petri dish at 25 °C. Immobilization conditions were statistically optimized by our group and described by Vasconcelos et al. [27].

#### 2.2.4. Production of BC+Papain Membrane

The methodology for immobilizing papain on the sterile wet BC membrane (~17 g wet weight; thickness of 4 mm) was similar to that used for immobilizing papain on the OxBC membrane (described in Section 2.2.3). Before immobilization, the purified BC membranes were sterilized by autoclaving at 121 °C and 1 atm for 15 min. The papain solution (2%, *w*/*v*) was prepared in 0.1 mol·L^−1^ citrate-phosphate buffer (pH = 7) and sterilized using a 0.22 μm PES filter. The sterile BC membrane was immersed in 25 mL (500 mg papain powder) of this solution in sterile polystyrene Petri dishes and incubated in a 45 °C oven for 24 h, all within a laminar flow cabinet. Post-immobilization, the membrane was washed with 25 mL of sterile PBS (0.1 mol·L^−1^, pH = 8) to remove the unbound enzyme and stored in a sterile Petri dish at 25 °C.

### 2.3. Characterizations

To assess enzymatic stability during storage, the BC+Papain and OxBC–Papain membranes were characterized. The water vapor transmission characteristics were evaluated for purified BC, OxBC, and OxBC–Papain membranes. Additionally, the absorption of aqueous solutions, hemocompatibility (including hemolysis and clotting time tests), cytotoxicity, and cell adhesion assays were conducted for purified BC and OxBC–Papain membranes. All methodologies are described below.

#### 2.3.1. Enzyme Stability during Storage

The enzymatic activity of the wet BC+Papain (without chemical immobilization of the enzyme) and OxBC–Papain membranes were monitored over 100 days of storage. The wet membranes with papain (6.7 cm in diameter) were aseptically packaged in sterile, sealed, and transparent unit packaging. The packages were stored in polystyrene boxes under controlled conditions of 25 °C, 85% air humidity, and no light.

The enzymatic activity of papain in the BC+Papain and OxBC–Papain membranes was measured after 29, 46, 63, and 100 days of storage using the protocols described in [32,33] with slight modifications. The methodology is described in detail in a previous work [27]. Briefly, the hydrolytic capacity of papain was measured by the amount of tyrosine released after casein hydrolysis. One unit of enzyme was defined as the amount that hydrolyzes casein to produce an absorbance equivalent to 1 μmol of tyrosine per minute. The percentage of relative activity was calculated using Equation (1). The papain hydrolytic activity assays in the membranes were performed in duplicate (*n* = 2) to calculate the mean and standard deviation for each storage period.

Relative activity (%) = (Papain activity after storage/Initial papain activity) × 100(1)

#### 2.3.2. Absorption of Aqueous Solution

The absorption capacity of aqueous solutions was determined using the gravimetric method [30,34]. The membranes, dried at 25 °C for 24 h, were cut into discs (16 mm in diameter and 1.0 mm thick), and their initial weights (W_dry_) were measured using an analytical balance (Analytical Plus AP250D, Ohaus, Pine Brook, NJ, USA). The membranes were then immersed in 10 mL of various saline solutions, including Simulated Body Fluid (SBF), physiological solution (0.9% NaCl, *w*/*v*), and phosphate-buffered saline (PBS), for 24, 48, and 72 h at 37 °C. After each immersion period, excess liquid was removed from the surface of the membranes by lightly pressing them with filter paper. The samples were then weighed to determine their final weights (W_wet_). The degree of swelling (DS), in percentage, of the purified BC and OxBC–Papain membranes was calculated using Equation (2). These experiments were conducted with at least five replications (*n* = 5) for each aqueous solution.

DS (%) = (W_wet_ − W_dry_)/W_dry_ × 100 (2)

#### 2.3.3. Water Vapor Transmission (WVT)

The water vapor transmission rate (WVTR) through the purified BC and OxBC–Papain membranes was determined by following ASTM International (E96/E96M-16, 2016) [35]. This test measures the amount of moisture permeating through the membrane over 24 h. The freeze-dried membranes were cut into discs (30 mm in diameter and 1.5 mm thick) and placed to seal the mouth of a cylindrical beaker (24 mm in diameter) containing 2 mL of deionized water. The samples were securely fixed and sealed with silicone to prevent moisture loss. The assembled system (cylindrical container with water + membrane) was initially weighed using an analytical balance (Marte AY220, Shimadzu, Barueri, SP, BR) and incubated at 37 °C in a vertical desiccator (ARSEC DCV-040) containing silica to maintain a relative humidity of 85%. Water evaporation through the membrane was determined by periodically weighing the system over 24 h. The decrease in the mass of the system indicated the loss of water through the membrane. The WVTR was calculated by the loss of water mass over 24 h divided by the surface area of the membrane covering the cylindrical container, as detailed in Equation (3). For each sample, nine systems (*n* = 9) were assembled and weighed to calculate the mean values and standard deviation.

WVTR (g/m^2^·24 h) = (W_initial_ − W_final_)/(π × R^2^) (3)

where W_initial_ represents the initial weight of the system (in g); W_final_ represents the final weight of the system after 24 h (in g); π represents the value of pi (π = 3.14159); and R represents the radius of the cylindrical beaker (1.2 × 10^−3^ m). Therefore, π × R^2^ = 4.524 × 10^−3^ m^2^, which represents the effective area of the water vapor permeability membrane.

#### 2.3.4. Hemolysis Test

Hemolytic activity was tested in Petri dishes using the technique based on blood agar diffusion, as described in [30]. The blood agar medium was prepared by mixing 5 mL of human blood (collected from healthy donors in vacuum collection tubes containing sodium citrate as an anticoagulant agent) with 95 mL of sterile nutrient agar, handled aseptically. Immediately, 25 mL of the mixture was transferred to a sterile polystyrene Petri dish (150 × 25 mm) in a laminar flow cabinet. After solidification, purified BC and OxBC–Papain membranes (14 mm in diameter and 4 mm thick) were placed in contact with the surface of the blood agar and incubated at 25 °C for 24 h. The positive control (hemolytic) was 200 μL of sterile saponin solution 10% (*w*/*v*) and the negative control (non-hemolytic) was 200 μL of sterile NaCl solution 0.9% (*w*/*v*). The assay was performed in duplicate for each plate, with three independent test plates used (*n* = 3).

#### 2.3.5. Clotting Time

The free hemoglobin methodology was used to evaluate the coagulation time when blood comes into contact with the surface of the wound dressing. This methodology was adapted from previous studies [36,37]. The wet membranes (purified BC and OxBC–Papain) were cut into discs with a 14 mm diameter and placed in a 24-well plate. Blood was previously collected from healthy donors. For each 2 mL of citrated blood, 400 μL of CaCl_2_ was added and homogenized. Immediately after preparing this solution, 50 μL of the blood was placed in contact with the surface of the samples and incubated at 37 °C for 15, 30, and 60 min. After the incubation period, 2 mL of deionized water was carefully added to each well (enough to cover the entire surface of the sample). This causes red blood cells that are not trapped in the thrombus to undergo hemolysis and release hemoglobin into the supernatant. The amount of free hemoglobin was inversely proportional to the thrombus formed. Subsequently, 100 μL of the supernatant was transferred to a 96-well plate. The free hemoglobin molecules were colorimetrically quantified by measuring absorbance at 540 nm using a SpectraMax i3x ELISA (Molecular Devices, San Jose, CA, USA). The negative control was performed by adding 2 mL of deionized water to 50 μL of citrated blood, representing the total free hemoglobin. The coagulation percentage was calculated by dividing the absorbance of samples by the absorbance of the negative control. Three independent tests were performed (*n* = 3), using one donor for each test, with five samples per condition.

#### 2.3.6. Cytotoxicity

The indirect method used L929 cells (mouse fibroblasts) and HaCat cells (human keratinocytes) for the cytotoxicity assay. To prepare the extracts, 1 mL of high-glucose Dulbecco’s Modified Eagle’s Medium (h-DMEM) was added to moist purified BC and OxBC–papain membranes (14 mm in diameter and 3 mm thick) and incubated for 24 h at 37 °C according to the guidelines of the International Organization of Standardization (ISO 10993-5:2009) [38]. After the incubation period, extracts were collected and supplemented with 10% (*v*/*v*) fetal bovine serum (FBS) and 1% (*v*/*v*) Penicillin-Streptomycin.

L929 and HaCat cells were cultured in h-DMEM containing 10% (*v*/*v*) FBS and 1% (*v*/*v*) Penicillin-Streptomycin. For HaCat cells, 1 mmol·L^−1^ sodium pyruvate was added to the supplemented h-DMEM. Viable cells were determined using the trypan blue exclusion method. In a 96-well plate, cells (density of 6 × 10^4^ cells/well) were incubated at 37 °C (5% CO_2_ and 95% air) for 24 h. Then, the supplemented h-DMEM was replaced with 100 μL of supplemented DMEM extract and incubated again at 37 °C for 24 h, as recommended by ISO 10993-5:2009. Metabolically active cells were measured using the fluorescent assay for the irreversible conversion of Resazurin (non-fluorescent blue) to Resorufin (fluorescent pink). The h-DMEM extract was removed from the wells, and 120 μL of AlamarBlue solution (1 mL of stock Resazurin solution (250 mg·L^−1^ in sterile PBS) and 9 mL of supplemented h-DMEM) was added, which was metabolized for 4 h under standard culture conditions. Subsequently, 100 μL of AlamarBlue solution metabolized by the cells was carefully mixed and transferred to a 96-well plate and measured using a microplate reader (SpectraMax i3x, Molecular Device, San Jose, CA, USA) in fluorescence mode (λ_excitation_ = 560 nm and λ_emission_ = 590 nm). Negative control cells were exposed only to supplemented h-DMEM, and their viability was set to 100% for the calculation of mean values and standard deviation. The percentage of metabolically active cells was calculated by dividing the sample’s fluorescence by the negative fluorescence of the control. The assay was conducted in triplicate across three independent experiments (*n* = 3). To maintain authenticity, cells were subcultured every two days when they reached more than 80% confluency, ensuring independent experiments.

#### 2.3.7. Cell Adhesion

Human dermal fibroblast cells (HDF) were used directly for the cytotoxicity assay. They were cultured in high-glucose Dulbecco’s modified Eagle medium (h-DMEM) containing 10% (*v*/*v*) fetal bovine serum (FBS) and 1% (*v*/*v*) Penicillin-Streptomycin. Wet sterile membranes of purified BC and OxBC–Papain (14 mm in diameter and 3.7–4 mm thick) were placed in non-adherent 24-well plates. A collagen matrix was used as a positive control for cell adhesion. In each well, 1 mL of viable HDF cells (density of 6 × 10^4^ cells/well), determined using the trypan blue exclusion method, was added to the surface of the membranes. The plate was incubated at 37 °C under an atmosphere of 5% CO_2_ and 95% air for 24 h according to the guidelines of the International Organization of Standardization (ISO 10993-5:2009) [38]. The h-DMEM was carefully removed from each well, and then the membranes were washed with sterile PBS.

The metabolically active cells adhered to the samples and the positive control was quantified using the AlamarBlue method, which involves the irreversible conversion of Resazurin (non-fluorescent blue) to Resorufin (fluorescent pink). An amount of 1 mL of AlamarBlue solution (1 mL of resazurin stock (250 mg·L^−1^ in sterile PBS) and 9 mL of supplemented h-DMEM) was metabolized for 4 h under standard culture conditions. Then, 100 μL of the metabolized AlamarBlue solution was transferred to a 96-well plate and measured in a microplate reader (SpectraMax i3x, Molecular Device, USA) in fluorescence mode (λ_excitation_ = 560 nm and λ_emission_ = 590 nm). The percentage of cells adhered to the membranes was calculated by dividing the samples’ fluorescence by the positive control’s fluorescence. The assay was conducted in triplicate across three independent experiments (*n* = 3). To maintain consistency, cells were subcultured every two days when they reached more than 80% confluency, ensuring independent experiments.

#### 2.3.8. Graphs and Statistical Analysis

All data presented in the graphs are expressed as mean ± standard deviation (SD). The graphs were created using OriginPro 2022 software (OriginLab Corporation, Northampton, MA, USA). An analysis of variance (ANOVA) was performed, and mean differences were compared using Tukey’s test (α = 0.05) with Statistica 10.0 software (StatSoft Inc., 2011, Tulsa, OK, USA).

## 3. Results

### 3.1. Effect of Storage on Papain Stability

**Figure 1:**
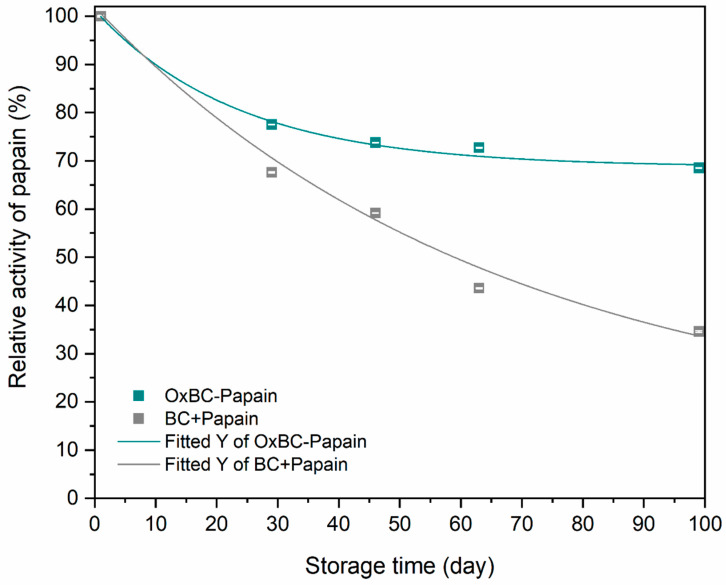
Relative enzymatic activity profile of papain in wet OxBC–Papain and BC+Papain membranes over 100 days of storage.

The stability of papain in the wet BC+Papain and OxBC–Papain membranes, stored aseptically for 100 days, was assessed. The relative activity of papain in both membrane types was compared, as shown in Figure 1. The corresponding enzyme activity values are detailed in Table 1. The membranes maintained their color throughout the storage period, indicating that the aseptic conditions during the preparation of the dressing were preserved. Additionally, the masses of BC+Papain and OxBC–Papain were kept practically constant, characterizing the absence of degradation of the membranes under storage conditions for 100 days.

In the first 29 days of storage, the relative proteolytic activity of papain in OxBC–papain was significantly higher than that in BC+Papain, as seen in Table 1. For OxBC–Papain, this represents a 22.5% reduction in its initial activity compared to a 32.4% reduction in the proteolytic activity of BC+Papain.

After 46 days of storage, the relative activity of papain in OxBC–Papain was similar to that observed at 29 days, showing only a 3% decrease over 17 days. In contrast, the BC+Papain membrane exhibited an 8.4% decrease in relative papain activity compared to the results from 29 days. These results indicate that the proteolytic stability of papain in membranes shows a distinct profile, with a gradual and more pronounced loss of proteolytic activity in BC+Papain.

Throughout the 63-day assay, the OxBC–Papain membrane maintained relatively constant proteolytic activity with values similar to those observed at 29 and 46 days of storage. In contrast, the BC+Papain membrane continued to experience a gradual decline in relative papain activity, showing a reduction of 56.4% compared to the initial activity measured for the material.

After 100 days of storage, a noticeable difference in papain stability was observed, as indicated by the varying results in relative papain activity. The OxBC–Papain membrane maintained its relative activity, while the BC+Papain membrane showed a significant decrease in proteolytic activity compared to its initial levels. Therefore, since the stabilization effect was more advantageous in OxBC, the wet OxBC–Papain and purified BC membranes were subjected to a detailed analysis to evaluate their in vitro physicochemical and biological properties, as presented below.

### 3.2. Degree of Swelling and Water Vapor Transmission

**Figure 2:**
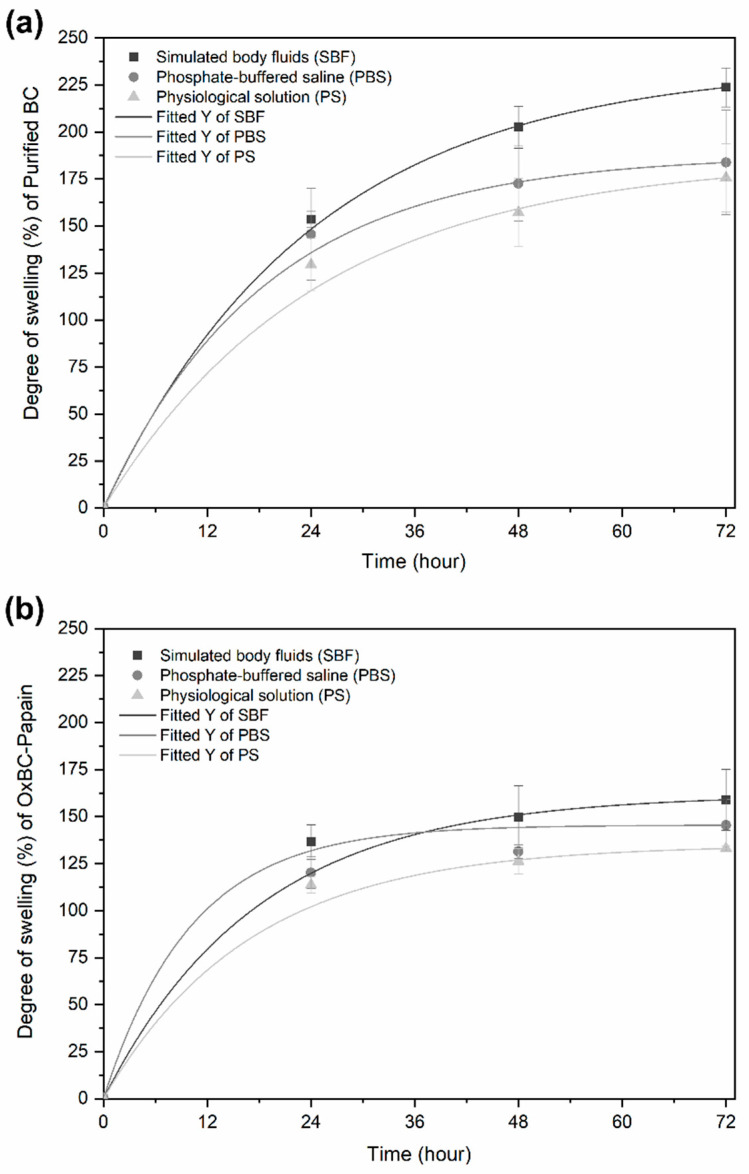
Profile of degree of swelling in aqueous solutions of membranes. (**a**) Purified BC and (**b**) OxBC–Papain, previously dried at 25 °C for 24 h.

Figure 2 presents the absorption profiles of the samples in three types of aqueous solutions: simulated body fluid (SBF), phosphate-buffered saline (PBS), and physiological solution (PS).

The degree of swelling (DS) quantifies how many times the membrane’s mass increases due to the absorption of an aqueous solution over 72 h. When comparing the swelling profiles, it becomes evident that the samples exhibited the same absorption trend for the aqueous solutions. Among these solutions, simulated body fluid (SBF) induced the highest swelling capacity for both membranes, followed by phosphate-buffered saline (PBS) and physiological saline (PS). This is due to the higher osmolarity of SBF, which favors osmotic pressure, inducing the swelling process in the membranes.

When comparing the membranes individually, purified BC exhibited a higher swelling profile than OxBC–Papain. This is supported by the DS values for SBF, which ranged from 150% to 220% after 72 h for purified BC. In contrast, the DS values for OxBC–Papain were lower, ranging from 140% to 160% over the same period. This represents an approximate 19% reduction in the swelling capacity of the OxBC–Papain membrane.

In the first 24 h, both membranes swelled rapidly due to their hydrophilic nature. For purified BC, this is attributed to the hydrophilic nature of cellulose. OxBC–Papain swelled due to the presence of hydroxyl and aldehyde groups (from OxBC) and hydroxyl and amino groups (from papain), which are hydrophilic and likely to form strong interactions with water molecules, favoring the absorption of aqueous solutions.

After 24 h, the swelling process slowed in both membranes. This suggests that the solutions permeate through the smaller pores of the membrane, gradually increasing the mass of the membranes until they reach full saturation. The DS values observed for the purified BC and OxBC–Papain membranes ranged between 100% and 900% of their initial mass in the aqueous solution, meeting the criteria for an ideal dressing [14]. The membranes did not show significant changes in thickness or diameter after the test, nor did they exhibit physical degradation (mass loss) after saturation with the tested aqueous solutions.

The water vapor transmission rate (WVTR) values for the freeze-dried purified BC, OxBC, and OxBC–Papain membranes were 3320 ± 219 g/m^2^·24 h, 2652 ± 53 g/m^2^·24 h, and 2678 ± 181 g/m^2^·24 h, respectively. A reduction of approximately 19% in water vapor permeability was observed when comparing BC with OxBC and OxBC–Papain membranes. This reduction in the WVTR value is primarily associated with a decrease in membrane porosity due to the oxidation process, which promoted nanofibril aggregation and the compaction of cellulose layers. The porosity of the freeze-dried BC and OxBC membranes was 57.4% and 36%, respectively, reflecting a 37% reduction [23].

Although papain is hydrophilic, its large macromolecule (approximately 23 kDa) may occupy the largest pores in the OxBC membrane, potentially blocking water vapor passage and reducing the WVTR value. However, the WVTR values for the freeze-dried OxBC and OxBC–Papain membranes did not differ significantly, suggesting that the physical barrier effect of papain had a minimal impact on water vapor permeation.

### 3.3. Hemocompatibility and Cell Assays

**Figure 3:**
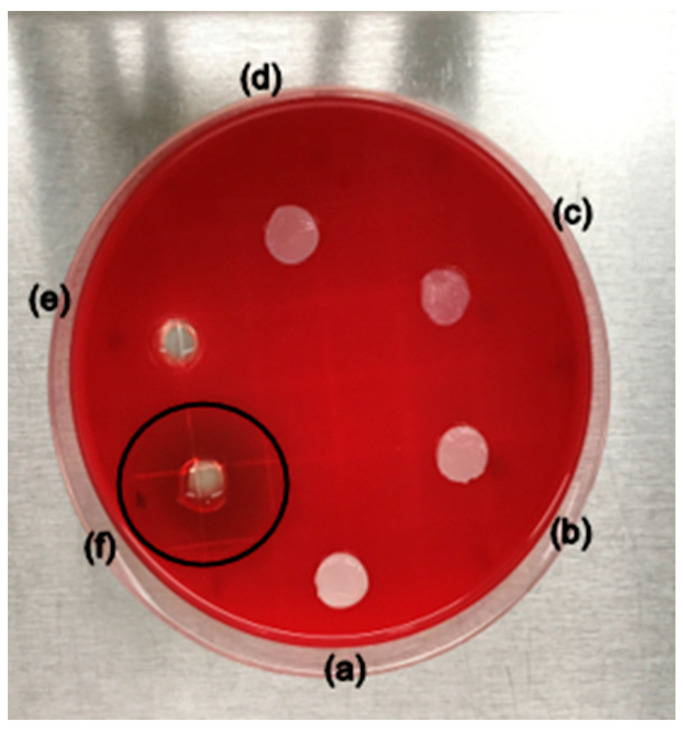
Qualitative hemolytic activity by diffusion on blood agar of wet membranes: (**a**,**b**) purified BC, (**c**,**d**) OxBC–Papain, (**e**) negative control (NaCl, 0.9% *w*/*v*), and (**f**) positive control (saponin, 10% *w*/*v*). The black circle indicates halo formation due to blood hemolysis.

Hemolytic properties were assessed to determine the suitability of purified BC and wet OxBC–Papain membranes as potential dressings. The qualitative results of the hemolysis test, performed on blood agar, showed that the wet membranes did not exhibit a hemolytic effect, as shown in Figure 3.

Hemolytic activity is indicated by a halo around the materials, which shows that erythrocytes are being prematurely destroyed. The positive control (saponin, 10% *w*/*v*) displayed a halo measuring 42 ± 3 mm, confirming hemolysis. In contrast, neither the purified BC nor OxBC–Papain membranes produced a halo, indicating that these materials do not induce hemolysis upon contact with blood. The enzyme release assay conducted in Franz cells revealed that approximately 60.2 mg·mL^−1^ of papain was released into the medium after 24 h, as reported by Vasconcelos et al. [27]. This amount was insufficient to cause red blood cell plasma membrane rupture. Moreover, when the membranes were removed from the blood agar surface, there was no change in the color of the medium.

The hemostatic properties of the purified BC and OxBC–Papain membranes were evaluated using the free hemoglobin (FH) method, which assesses the blood clotting capacity. The relationship between the amount of FH and the coagulative capacity is inversely proportional. Thus, the lower the percentage of FH, the higher the coagulative capacity of the dressing.

**Figure 4:**
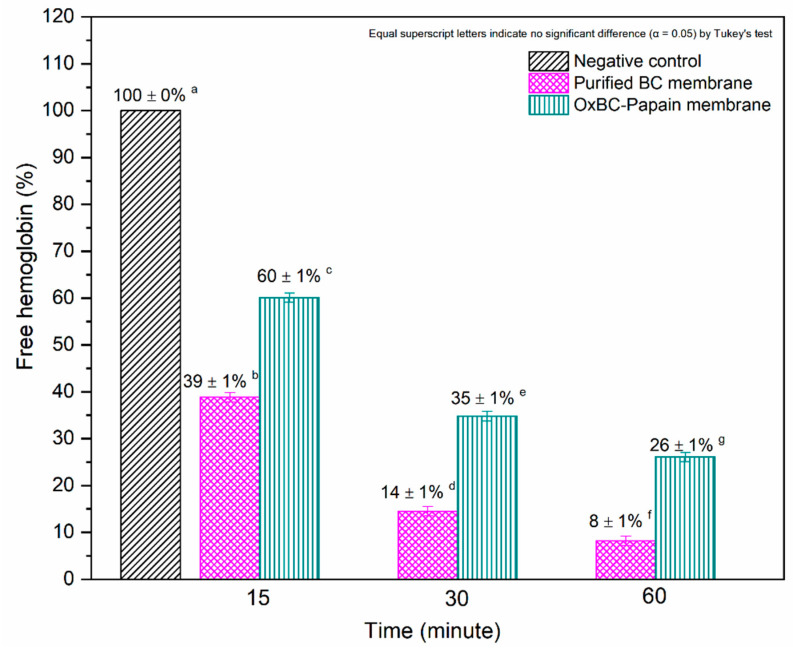
Percentage of free hemoglobin (FH) after 15, 30, and 60 min of contact with purified BC and wet OxBC–Papain membranes. Equal superscript letters indicate no significant difference (α = 0.05), as determined by Tukey’s test.

The results demonstrate that both membranes significantly reduced FH, indicating a favorable coagulation process. The FH values decreased over time while being in contact with the membranes, showing a hemostatic profile with human blood. Figure 4 shows the relationship between the percentage of FH and the contact time with the membranes, with statistically significant differences being observed between the membranes and the negative control.

**Figure 5:**
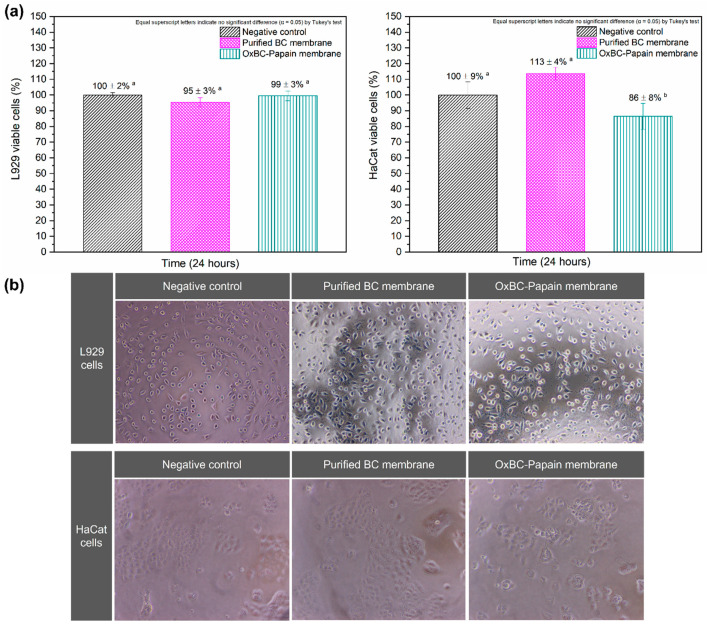
Results showing (**a**) cell viability of fibroblasts (L929 cells) and keratinocytes (HaCat cells), and (**b**) optical micrographs (100× magnification, 2 mm) of wells containing respective cells after 24 h, obtained from indirect cytotoxicity assays of purified BC and wet OxBC–papain membranes. Equal superscript letters indicate no significant difference (α = 0.05), as determined by Tukey’s test.

Indirect cytotoxicity assays were conducted to evaluate the biocompatibility of purified BC and wet OxBC–Papain membranes, as well as the behavior of papain physically immobilized on the wet OxBC membrane, using fibroblasts (L929) and keratinocytes (HaCaT). The cytotoxicity results are presented in Figure 5a. Figure 5b displays optical micrographs of the wells containing the respective cells after 24 h, showing that both the L929 and HaCaT cells maintained their characteristic morphology, similar to the control group, indicating viability without apoptosis or autophagy.

The purified BC and OxBC–Papain membranes showed high cell viability for the L929 cells, with percentages of 95% and 99%, respectively, without significant differences. For the HaCaT cells, the viability was 113% for purified BC and 86% for OxBC–Papain, with a statistically significant difference attributed to the action of papain, which affects keratin, a structural protein produced by keratinocytes. However, the metabolic viability results of the L929 and HaCat cells were not significantly different from the negative control of the assay.

**Figure 6:**
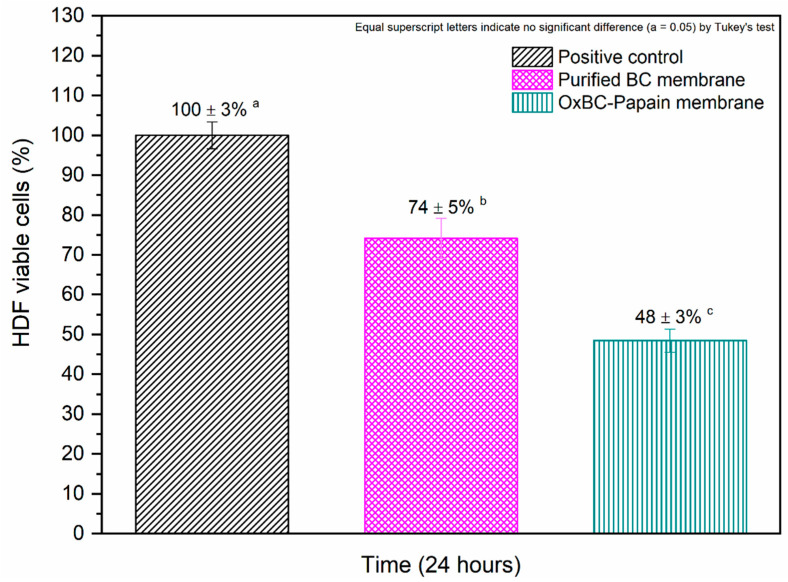
The viability of human dermal fibroblast (HDF cells) cultured directly on the surface of purified BC and wet OxBC–papain membranes after 24 h. Equal superscript letters indicate no significant difference (α = 0.05), as determined by Tukey’s test.

The cell adhesion assay (direct cytotoxicity) was performed using human dermal fibroblasts (HDF cells) to assess the properties of OxBC–Papain and the behavior of chemically immobilized papain. The results of the percentage of metabolically active HDF cells that adhered to the wet membranes of purified BC and OxBC–Papain are shown in Figure 6.

The results were statistically significant compared to the assay’s positive control. For purified BC, the percentage of cells that adhered to the membrane surface was greater than 70% although lower than that observed in the collagen matrix (a membrane with known adhesive properties). In contrast, the percentage of HDF cell adhesion on the OxBC-Papain membrane was less than 50% compared to the positive control membrane. This reduction in cell adhesion on the OxBC–Papain membrane correlates with the presence of active papain, which cleaves glycoproteins involved in cell adhesion.

## 4. Discussion

### 4.1. Papain in Wet Oxidized Bacterial Cellulose Membranes: Demonstrated Benefits

The periodic evaluation of papain activity in wet OxBC and BC membranes demonstrates that the oxidized membrane offers significant advantages for papain immobilization. The increased stability of papain in OxBC–Papain compared to BC+Papain is attributed to the dual physical and chemical immobilization processes provided by the OxBC membrane. This stabilization ensures prolonged enzymatic activity and effectiveness in wound debridement, addressing chronic wounds more efficiently.

The initial higher proteolytic activity of papain in OxBC–Papain and its more gradual decline over time suggest that the chemical immobilization of papain in OxBC maintains enzymatic activity better under storage conditions. The comparative analysis with BC+Papain, which showed a more rapid loss of proteolytic activity, supports the benefit of using oxidized membranes for enhanced enzyme stability.

The immobilization mechanism of papain on OxBC involves both physical adsorption and strong covalent bonding. Physically immobilized papain is released into the wound initially, but its activity may decrease due to environmental conditions. Chemically immobilized papain, with its more stable structure, continues to act effectively on the wound surface, providing sustained biocatalytic action and reducing the need for frequent dressing changes.

Previous studies have demonstrated that the OxBC membrane can incorporate a higher amount of papain compared to the BC membrane, contributing to enhanced healing processes [27]. Specifically, the OxBC membrane can accommodate approximately 264.5 mg of papain, encompassing both physically adsorbed and covalently immobilized forms, as indicated by the immobilization yield (IY = 52.9%) reported by Vasconcelos et al. [27].

The purified wet BC membrane, even in the absence of papain, exhibits promising attributes, including 57.4% porosity and 5.3 ± 0.8 MPa mechanical strength [23], which underscore its potential as an effective therapeutic wound dressing. Additionally, the OxBC–Papain membrane benefits from the stabilization of papain, enhancing its therapeutic efficacy. Together, these features highlight the potential of both the purified BC and wet OxBC–Papain membranes as suitable therapeutic options. The discussion of the results provides scientific evidence supporting their use in different phases of the healing process: the purified BC membrane as a passive dressing and the OxBC–Papain membrane as a bioactive dressing.

### 4.2. Assessment of Physicochemical Properties

The results show that purified BC membranes have a higher swelling capacity compared to OxBC–Papain membranes. This difference can be attributed to the lower porosity of the OxBC membrane, resulting from the oxidation process, as reported by Vasconcelos et al. [23]. Despite the reduction in swelling, the incorporation of papain into the OxBC membrane did not compromise its use as a dressing for chronic wounds.

In addition to porosity, the oxidation process with the periodate ion (IO_4_^−^) of BC promotes the replacement of hydroxyl groups by aldehyde groups, according to the mechanism demonstrated in the literature [23,39,40]. Aldehyde groups are less hydrophilic than hydroxyl groups due to differences in their ability to form hydrogen bonds with water molecules in which aldehyde groups can only form one hydrogen bond (as an acceptor). In contrast, hydroxyl groups can form two hydrogen bonds (one as a donor and one as an acceptor), significantly increasing their interaction with water.

The rapid swelling observed in the first 24 h is beneficial for wound healing, as excess exudate in the wound slows cell proliferation and contributes to infection due to bacteria on the skin surface [41]. Slower absorption after 24 h helps maintain a moist environment, preventing local dehydration and protecting the wound against bacterial invasion. This also regulates the temperature at the injury site and promotes the painless removal of the dressing during changes.

The water absorbed by the OxBC–Papain membrane acts as a nucleophile in the proteolytic action of chemically immobilized papain, attacking the carbonyl carbon of peptide bonds and leading to their cleavage. Additionally, intrinsic water in the membrane facilitates the release of physically adsorbed papain and helps maintain its proper three-dimensional conformation. This ensures that the active site remains accessible and functional for debriding necrotic and purulent tissue.

The stability of the membranes, as evidenced by the lack of significant changes in the thickness, diameter, or mass loss after saturation, is advantageous for their application as skin dressings. It ensures complete wound protection during use.

The results demonstrate that the oxidation process of the BC membrane had a more significant effect on reducing the WVTR than the incorporation of papain into the OxBC membrane. This finding is consistent with previous studies, such as those by Lin et al. [42], who reported a reduction in the WVTR for BC membranes with incorporated chitosan. In their study, the WVTR value for a freeze-dried BC membrane was 1503 g/m^2^·24 h, and after the incorporation of chitosan, it decreased to 1460 g/m^2^·24 h.

Our WVTR values exceed those reported by Lin et al. [42] and fall within the ideal range according to the World Health Organization, which states that WVTR values for ideal dressings range from 2000 to 2500 g/m^2^·24 h [14]. For adequate performance, WVTR values must be lower than or close to the damaged skin to avoid dehydration. Normal skin presents a WVTR value of 204 g/m^2^·24 h [42], whereas injured/inflamed skin tends to lose more moisture, with values ranging from 279 g/m^2^·24 h for first-degree burns to 5138 g/m^2^·24 h for chronic skin wounds, respectively [43].

Currently, dressings available with high water vapor transmissibility are already on the market, such as Opsite Post-op^®^ and Hydrocoll^®^, which provide a WVTR of 3000 g/m^2^·24 h. These products are recommended for wounds in the inflammatory phase that have a shiny and moist appearance with high exudate production.

The physicochemical properties of purified BC and OxBC–Papain membranes suggest their distinct applications. Purified BC demonstrates higher WVTR results than commercial high-performance dressings, making it suitable for therapeutic use in wounds in the inflammatory phase. In contrast, OxBC–Papain exhibits a lower WVTR than commercial permeable dressings but remains within the ideal range for wound dressings. This membrane provides a sufficient gas exchange capacity and exhibits debriding properties, making it suitable for therapeutic use in wounds with necrotic tissue.

A lower WVTR, combined with the DS in dressings containing bioactive compounds, is crucial for maintaining wound hydration, facilitating the rapid delivery of bioactive compounds, and minimizing patient discomfort during dressing changes. For enzymatic debridement, such as with OxBC–Papain, controlled humidity prevents the wet dressing from drying out under the wound, as the enzyme relies on water for effective proteolysis [28,32,44].

In conclusion, while purified BC is suitable for wounds in the inflammatory phase due to its higher WVTR, OxBC–Papain is ideal for wounds requiring enzymatic debridement and controlled humidity.

### 4.3. Assessment of Biological Properties (In Vitro)

The hemocompatibility of a dressing can be assessed through its hemolytic properties. In healthy humans, the volumetric fraction of erythrocytes (red blood cells) in the blood is approximately 48% [45]. The preservation of these cells at the injury site directs biochemical stimuli produced by macrophages toward angiogenesis. Increased tissue oxygenation facilitated by active hemoglobin enhances fibroplasia and, consequently, fibroblast proliferation [46]. As the mitotic activity of these cells rises, the damaged extracellular matrix is restored, providing a scaffold for cellular regeneration and new blood vessel formation. As wound healing progresses, capillaries formed at the site undergo apoptosis, resulting in a shift from the vibrant red of granulation tissue to a paler hue [47]. Thus, if a wound dressing induces hemolysis, it hampers stimulus initiation due to oxygen deprivation, impairing tissue growth and, consequently, its repair.

The hemostatic evaluation revealed that purified BC has a higher coagulation capacity compared to OxBC–Papain. This difference can be attributed to two interrelated factors. The first factor concerns the porosity of the membranes, with OxBC–Papain being affected by the oxidation process [23]. This is directly related to the structural properties of the materials, which influence the intrinsic coagulation process upon contact with a wound. Reduced porosity in OxBC–Papain hinders platelet aggregation between the oxidized bacterial cellulose nanofibrils, impairing thrombus formation and, consequently, hemostasis [48,49,50]. The second factor is exclusively related to the interference of papain in the tissue coagulation cascade, known as extrinsic coagulation. Papain, a protease, acts by inhibiting the conversion of prothrombin into thrombin, directly affecting the transformation of fibrinogen into fibrin [51]. This process inhibits platelet aggregation and fibrin polymerization, making clot formation difficult [52]. In addition to the extrinsic coagulation mechanism, papain is an endopeptidase that contains cysteine in its active site, allowing it to cause the disintegration of fibrin into soluble products, a process known as fibrinolysis [51].

Coagulation is a simultaneous process that occurs through two distinct pathways: the contact pathway, known as intrinsic coagulation, and the biochemical pathway, known as extrinsic coagulation. Firstly, the physical structure of the skin-covering materials plays a crucial role in triggering the intrinsic coagulation process. In this sense, the purified wet BC membrane has a greater influence as it mainly acts through direct contact due to its porosity and the absence of papain. Conversely, in the biochemical pathway, the presence of papain exerts a greater influence on the extrinsic coagulation process. Therefore, the biochemical factor is more predominant in the wet OxBC–Papain membrane due to the presence of the enzyme. Fibrinolytic and blood clot lysis activities are expected characteristics of papain in a dose-dependent manner and also serve as confirmation of its proteolytic activity [51]. Thus, the results obtained for OxBC–Papain prove its biocatalytic activity; however, it acts at doses promoting clot formation.

In the literature, it is reported that papain does not exhibit substance specificity regarding the antigens that classify blood groups [53]. Therefore, the patient’s blood types (A, B, and O) did not affect the hemocompatibility of the wet OxBC–Papain membrane.

The preservation of high cell viability in both purified BC and wet OxBC–Papain membranes indicates their biocompatibility and effectiveness as potential skin dressings. The indirect cytotoxicity assays showed that both types of membranes maintained high metabolic activity in fibroblasts (L929) and keratinocytes (HaCaT), essential for effective wound healing.

In terms of papain release, approximately 53% of the papain adsorbed on the OxBC membrane was released after 24 h, with an immobilization yield (IY) of 52.9% and a proteolytic activity recovery (AR) of 93.3%, according to what was reported by Vasconcelos et al. [27]. As previously mentioned, the in vitro release assay using Franz cells showed a papain concentration of 60.2 mg/mL after 24 h. This concentration matches the level observed in cell culture extracts, indicating that the amount of enzyme released only significantly affected the viability of the HaCaT cells.

According to the literature, the relative cell growth rate for materials with biomedical applications must be greater than 70–80% [42,54]. Therefore, the membranes proposed in this work meet this requirement, and they are considered cytocompatible (in vitro) with epidermal tissue. Additionally, as reported by Vasconcelos et al. [23], the oxidation of BC with sodium periodate did not exhibit a cytotoxic profile for L929 (98 ± 2%) or HaCaT cells, showing a percentage of metabolically active cells of 89 ± 7%. These results indicate that the chemical modification process in the wet BC membrane did not alter its cellular properties, maintaining the cytocompatibility of the material.

BC is widely recognized in the literature as a biocompatible material [3,55,56,57]. However, the low cytotoxicity of papain-functionalized OxBC is particularly valuable for scientific studies and, more importantly, for its application as a wound dressing. The cytocompatibility of membranes biofunctionalized with papain has also been reported in the literature [20,29,30,58].

The distinct cell adhesion profiles of the membranes can be attributed to differences in their chemical structures and surface charges, which affect molecular and electrostatic interactions with the glycoproteins present in the cell membrane. Collagen, for example, has adhesion proteins (such as fibronectin and laminin) and amino groups (positively charged), which favor this process. Cell adhesion on BC membranes is primarily promoted by matrix topography rather than biochemical pathways due to the absence of active sites for binding to integrins and other cell surface receptors present in the extracellular matrix (ECM) [59,60]. Regardless of the biochemistry of cell adhesion, substrate topography directly affects the ability of cells to adhere, orient, migrate, and produce organized cytoskeletal arrangements [61,62]. In this context, BC exhibits a topography similar to that of basement membranes, with pores and fibers in nanometric dimensions, facilitating cell adhesion on its surface [61], as observed in our study. Moreover, due to the similarity of its nanostructure and morphology to collagen, bacterial cellulose is a promising option for use in cell support and immobilization [63,64]. The adhesion values of L929 cells to the BC membrane ranged between 50% and 70%, with polystyrene material being used as the positive control, as reported by [42,65]. This variation may be attributed to the membrane’s topography.

Regarding the OxBC–Papain membrane, the reduction in the adhesion capacity of HDF cells is associated with the presence of active papain in the membrane, which, as a protease, cleaves the glycoproteins responsible for the cell–cell and cell–biomaterial bonds, specifically in the carbonyl region between the amino acids lysine and arginine. The in vitro results show that papain exerts a trypsin-like effect, releasing cells from the surface of flasks and plates [20,58]. However, regardless of the concentration and exposure time, it was found that papain does not modify the metabolism of these cells, ensuring the synthesis of collagen and elastin by skin fibroblasts [66]. Furthermore, the degradation of ECM proteins by proteolytic enzymes in response to wounding may induce the local release of these growth factors from their insoluble anchorage, thereby modulating the wound healing process [60]. This result is promising as it confirms the proteolytic activity of chemically immobilized papain, thus demonstrating the effectiveness of the enzyme’s active sites in biochemically interacting with proteins and biomolecules within the tissue, especially necrotic tissue. Consequently, papain facilitates debridement when in contact with the surface of the lesion without affecting collagen production by fibroblasts.

Unlike skin substitutes, an ideal dressing should not interact directly with the cells of the wound bed to prevent new tissue from adhering to the dressing [67]. This ensures that scar tissue will not be damaged during dressing changes. In this sense, the OxBC–Papain membrane meets the requirements for promoting the debridement of chronic wounds without causing damage to newly formed tissue.

### 4.4. Compilation of OxBC–Papain Properties

The OxBC–Papain wet membrane developed by our research group demonstrated favorable structural and physicochemical properties, including good water vapor transmission, swelling capacity, and biocompatibility with fibroblasts and keratinocytes. It showed non-cytotoxic and non-hemolytic effects, a promising coagulative capacity, and low cell adhesion, making it effective for the debridement of cutaneous wounds without damaging new tissue. Table 2 summarizes these observed OxBC–papain properties. Furthermore, the purified wet BC membranes exhibited structural, physicochemical, and biological properties, making them suitable for use as passive dressings in the final stages of healing (as illustrated in the graphical abstract). Both types of membranes maintain adequate moisture, prevent tissue drying, support epithelial cell proliferation, and promote minimal adhesion to the scar tissue.

## 5. Conclusions

The results compiled for the OxBC–Papain wet membrane underscore its potential as an advanced therapeutic dressing made from natural biomolecules, specifically papain. This membrane exhibits essential properties for the effective treatment of chronic wounds requiring enzymatic debridement. Both purified BC and OxBC–Papain membranes maintain adequate moisture, absorb excess exudate, promote gas exchange, have non-hemolytic effects and hemostatic capacity, are cytocompatible, and do not adhere to tissue.

The promising data presented in this paper encourage further investigation, particularly focusing on the epithelialization process of damaged tissue in in vivo models, as well as the practical application of these membranes in clinical settings. Such step-by-step studies could confirm their potential to provide comprehensive and efficient wound coverage, reducing the treatment time, minimizing the frequency of dressing changes, and consequently decreasing wound care costs.

## 6. Patent

This work is under evaluation by the National Institute of Intellectual Property (process number: BR 10 2022 016857 1).

## Figures and Tables

**Table 1:** Relative papain activity values (in percentage) for wet BC+Papain and OxBC–Papain membranes after 29, 46, 63, and 100 days of storage.

Storage Time (Days)	Relative Papain Activity (%)	
	BC+Papain	OxBC–Papain
29	67.6 ± 0.2 ^c^	77.5 ± 0.2 ^a^
46	59.2 ± 0.1 ^d^	73.8 ± 0.4 ^a^
63	43.6 ± 0.1 ^e^	72.7 ± 0.7 ^a^
100	34.6 ± 0.1 ^f^	68.5 ± 0.2 ^b^

Equal superscript letters indicate no significant difference (α = 0.05), as determined by Tukey’s test.

**Table 2:** The physicochemical, mechanical, structural, and in vitro biological properties of the wet OxBC–papain membrane.

Characterization Technique	Properties of Wet OxBC–Papain Membrane
Digital caliper (diameter × thickness)	67 × 3.7 mm [23]
Infrared moisture analyzers	Approximately 95–97%
Scanning electron microscopy (SEM)	Porosity gradient along the thickness of the membrane, with average nanofibrils of 212 nm in size [23]
Porosity (by BET)	Porosity of 36.1% [23]
Mechanical test (Young’s modulus)	Flexible wet membrane, with Young’s modulus of 1.7 MPa and deformation at rupture of 31% [23]
Physically adsorbed papain release profile (in vitro; Franz cells)	53% papain released under conditions simulating those of skin in homeostasis after 72 h [27]
Water vapor transmission rate (WVTR)	2678 g/m^2^; 24 h (freeze-dried material)
Hemocompatibility (in vitro)	Non-hemolytic and homeostatic behavior (26.1% clotting capacity after 1 h)
Cytocompatibility (in vitro)	Cell viability of 99% and 86% for L929 and HaCat, respectively
Cell adhesion (in vitro)	Non-adherent behavior (HDF viability of 48% on the surface)

## Data Availability

The raw data supporting the conclusions of this article will be made available by the authors on request.
